# Genome Sequence of Colistin-Resistant Escherichia coli CLR8, Isolated from the Feces of Larus argentatus

**DOI:** 10.1128/MRA.00533-20

**Published:** 2020-07-09

**Authors:** Takehiko Kenzaka

**Affiliations:** a Environmental Science and Microbiology, Faculty of Pharmacy, Osaka Ohtani University, Tondabayashi, Japan; University of Maryland School of Medicine

## Abstract

Migratory birds are potential vehicles of antibiotic-resistant bacteria. In this study, a colistin-resistant Escherichia coli strain, CLR8, was isolated from the feces of Larus argentatus. The draft genome sequence of the strain indicated that it hosts *mcr-1*, which is a plasmid-mediated colistin resistance gene.

## ANNOUNCEMENT

Owing to their ability to migrate long distances within short periods, migratory birds are a potential source of antibiotic-resistant bacteria (1–5). The European herring gull (Larus argentatus) breeds in the northernmost regions of Europe and Asia (6). In late summer, *L. argentatus* leaves its breeding grounds to arrive in autumn at its wintering grounds across Europe and Asia. This species mainly lives in coastal and near-coastal areas (7).

Colistin is one of the select few critical antibacterial drugs that are used for treating multidrug-resistant bacteria; it is listed as the last-resort antibiotic in the essential medicines list defined by WHO. The mobilized colistin resistance gene *mcr-1* was first identified in an Escherichia coli plasmid in 2011 (8). Since its discovery, *mcr* genes have globally disseminated, resulting in a significant public health threat (9).

In November 2017, fresh feces samples from a population of approximately 20 wild *L. argentatus* gulls were collected on a breakwater at a stopover point along their migration route, in Bekkaicho, Hokkaido, Japan. Partial sequencing of the gene encoding the NADH dehydrogenase subunit 2, which was isolated from the feces samples, confirmed the source to be *L. argentatus*. The colistin-resistant E. coli strain CLR8 was isolated using CHROMagar ECC medium (Kanto Chemical Co., Inc., Tokyo, Japan) containing colistin at 37°C for 24 h. CLR8 showed an MIC of 8 mg liter^−1^ for colistin. Genomic DNA was extracted using a QIAamp DNA minikit (Qiagen, Germany) from overnight cultures grown in 10 ml of LB broth at 37°C.

Genome sequencing was performed with a 20-kb SMRTbell library (PacBio DNA/polymerase binding kit P6) on the RS II sequencing platform at Macrogen Corp. (Kyoto, Japan). The PacBio sequence reads were filtered and assembled *de novo* using RS_HGAP Assembly v3.0. Default parameters were used for all software. A total of 136,406 reads that passed the filtering step with an *N*_50_ length of 9,733 bp were assembled. The assembly generated 11 scaffolds with an input read sequencing depth ranging from 78× to 109×. The scaffold *N*_50_ value was 776,242 bp. The NCBI Prokaryotic Genome Annotation Pipeline v4.11 was used to annotate the draft genome (10). The genome is 5.25 Mbp, with a G+C content of 50.05%. Additionally, the strain carries two plasmids, which were incomplete (229 and 101 kbp). The draft genome contains 4,834 protein-coding sequences, 90 tRNAs, 8 5S rRNAs, 8 16S rRNAs, and 8 23S rRNAs.

The resistance-associated genes were predicted with the ResFinder v3.2 server (https://cge.cbs.dtu.dk/services/ResFinder/) (11) with a nucleotide identity threshold of 90% and a minimum query length of 60%. Strain CLR8 harbored the trimethoprim resistance gene (*dfrA7*), the beta-lactam resistance gene (*bla*_CTX-M-65_), and the *mcr-1* gene at 100% nucleotide identity. Moreover, CLR8 also harbored the genes for the multidrug efflux transport protein (*mdfA*) and phenicol resistance (*floR*) at 98.2% nucleotide identity.

### Data availability.

The draft genome sequence of E. coli strain CLR8 has been deposited in GenBank under accession number JAAKGE000000000. The raw reads can be found in the SRA under accession number SRR9648576.
